# Persistent Eustachian Valve Causing Abdominal and Pelvic Venous System Dilation in a 44-Year-Old Male: A Case Report

**DOI:** 10.7759/cureus.60994

**Published:** 2024-05-24

**Authors:** Martin Nguyen, Christopher Gross, Jackie Buttafuoco, Sarah Ansari, Harpreet Nagi, David Maki

**Affiliations:** 1 Radiology, West Virginia School of Osteopathic Medicine, Lewisburg, USA; 2 Clinical Sciences, West Virginia School of Osteopathic Medicine, Lewisburg, USA; 3 Radiology, Clinical Sciences, West Virginia School of Osteopathic Medicine, Lewisburg, USA

**Keywords:** prominent eustachian valve, dilated inferior vena cava, pelvic venous system, abdominal venous system, persistent eustachian valve

## Abstract

This case report presents a rare incidence of a persistent Eustachian valve (EV) causing notable venous dilation in the abdominal and pelvic regions of a 44-year-old healthy male. Initially presenting with left flank pain, diagnostic evaluations identified a 4.8-mm calculus in the distal left ureterovesical junction. Incidentally, imaging also revealed unexplained venous distensions, subsequently attributed to a prominent EV obstructing the inferior vena cava (IVC). The EV, an embryological structure in fetal circulation that helps divert blood from the IVC to the left atrium via the foramen ovale, typically regresses postnatally. Its persistence into adulthood is uncommon and often does not necessitate intervention. However, a persistent EV is often associated with other cardiac findings, especially a patent foramen ovale (PFO) of an atrial septal defect (ASD). There were some reports demonstrating that persistent EV may play a role in an increased risk of paradoxical cerebral embolism in such cases. Therefore, the case underscores the importance of considering such embryological remnants in the differential diagnoses of unexplained venous distension and cryptogenic stroke. It also highlighted the need for a personalized approach to management, especially during the preparation phase before interventional procedures, such as an ASD closure, to minimize the risks during the operation. Furthermore, it also contributed to a broader understanding of the clinical implications of persistent embryological structures and emphasized the value of meticulous diagnostic processes in identifying the underlying causes of observed anomalies.

## Introduction

The Eustachian valve (EV) exists in the superior portion of the inferior vena cava (IVC), close to the junction of the IVC and the right atrium (RA). In fetal circulation, the EV plays an important role in diverting the blood flow towards the foramen ovale, thus bypassing the immature pulmonary circulation [1]. After birth, the foramen ovale is spontaneously closed, and the EV has no further specific function. Thus, it tends to regress and disappear during adulthood. The prevalence of persistent EV varied greatly among different studies. In a study of 1,100 patients investigated by transoesophageal echocardiography, Marek et. al. [2] reported the prevalence of persistent EV to be 4.2% (male:female ratio = 1.1:1). In a study of 153 children, Limacher et. al. [3] reported that 70% of them had persistent EV.

In the majority of cases, persistent EV is a benign finding and requires no further intervention. However, EV can lead to serious complications such as endocarditis and cyanosis during the neonatal period or later in life [1,4]. In this case, we present a previously healthy 44-year-old male presenting with left flank pain. During the imaging workup, in addition to a renal calculus, it was incidentally found that there was a remarkable distention of the abdominal venous system, together with the presence of a prominent EV in the RA.

## Case presentation

**Figure 1:**
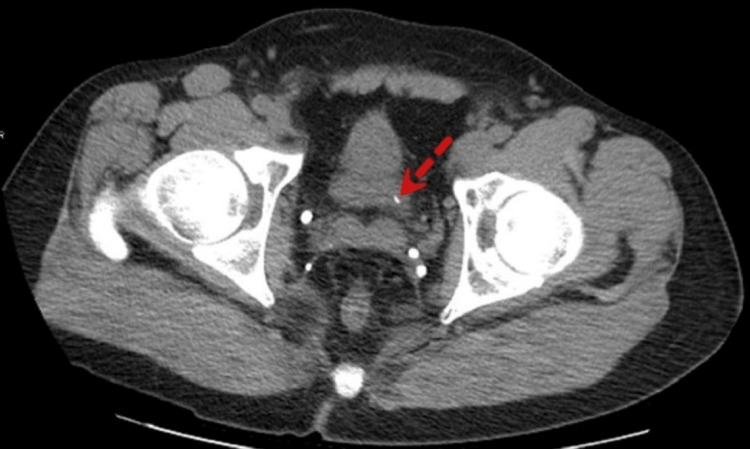
A renal stone (red dashed arrow) was detected in the left ureterovesical junction (computed tomography without contrast, axial view).

**Figure 2:**
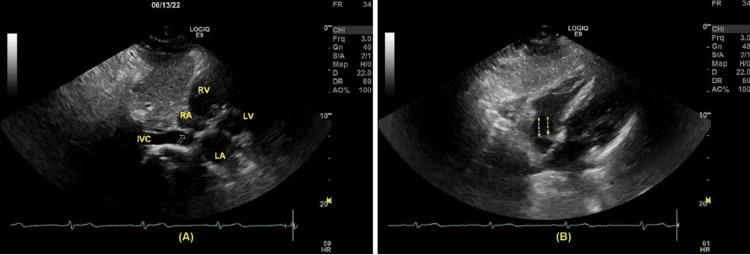
(A) The long axis of subcostal view demonstrated a persistent Eustachian valve (yellow arrow) in the right atrium; (B) The short axis of subcostal view demonstrated an Eustachian valve from a different angle (yellow dotted arrows). RA: right atrium, RV: right ventricle, LA: left atrium, LV: left ventricle, and IVC: inferior vena cana.

A 44-year-old male presents with left flank pain two weeks after passing a renal calculus. He did not have other symptoms, including chest pain, fatigue, or fever. A computed tomography (CT) scan of the abdomen was indicated for a renal calculus. A small stone (4.8 mm) was found at the left ureterovesical junction (Figure 1). Mild dilation of the left ureter and left hydronephrosis were demonstrated. Given the progressive nature of the patient’s symptoms, a transthoracic echocardiogram (TTE) was performed. No valvular abnormalities were detected, and the ejection fraction (EF) was 65%. The diastolic function of the left ventricle (LV) was within the normal range. The left atrium (LA) was mildly dilated, with no detectable mass inside. There was a structure in the RA consistent with a persistent EV (Figure 2). No right ventricular (RV) inflow obstruction was demonstrated. There was a trace of tricuspid regurgitation, and the RV function, as well as the estimated pulmonary artery systolic pressure, were within normal range.

**Figure 3:**
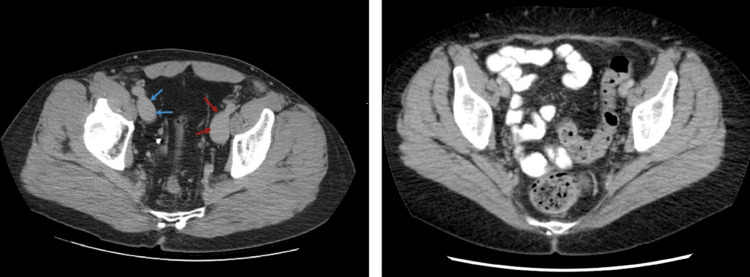
(Left) Dilated left (red arrows) and right (blue arrows) iliac vein (red arrows) in our patient; (Right) computed tomography at the same level in a normal person for comparison. (Right panel): Case courtesy of Andrew Dixon [5]. Permission was obtained from Radiopaedia.org.

**Figure 4:**
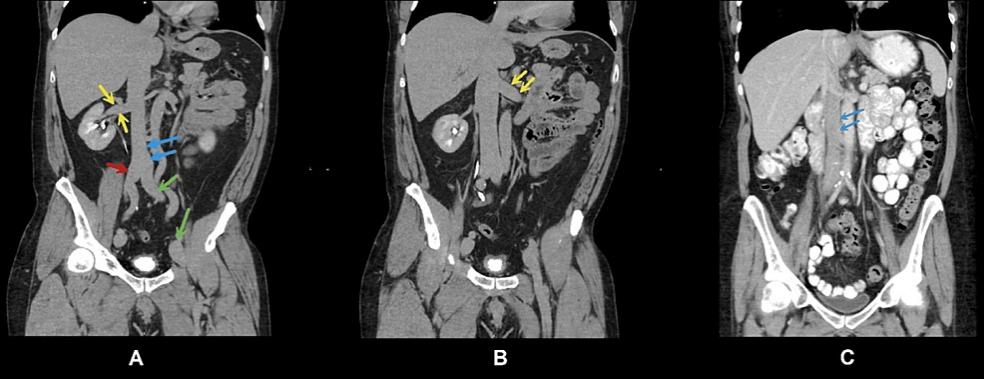
(A) and (B) panels are from our patients: (A) Dilated inferior vena cava (blue arrows), right renal vein (yellow arrows), right common iliac vein (red arrow), left common iliac vein (green arrows); (B) Dilated left renal vein (yellow arrows); (C) Computed tomography of a normal inferior vena cava in a healthy person for comparison. (Panel C) Case courtesy of Andrew Dixon [5]. Permission was obtained from Radiopaedia.org.

It was incidentally revealed that there was marked distension of the abdominal and pelvic venous vasculature. IVC, renal veins, and iliac veins were remarkably distended to the level of femoral veins (Figures 3, 4). The IVC diameter was recorded to be 3.8 cm. Although most cases of persistent EV are benign, serious complications can develop. In another visit two months later at another hospital for vascular surgery consultation, he complained about persistent fatigue, dyspnea, and chest tightness upon exertion, as well as a sensation of abdominal fullness. These symptoms have progressively worsened and are now impairing his ability to exercise. However, it was decided that surgical and medical interventions would not be necessary.

## Discussion

The persistence of sinus venosus valves results in EV and thebesian valve (ThV) [6]. In fetal circulation, the EV helps prevent regurgitation of blood into the IVC, while the ThV prevents blood from going back into the coronary sinus during atrial systole [7]. In adulthood, the EV is an embryologic remnant of the valve of the IVC [8]. There is also considerable variability regarding the size, shape, thickness, and texture of persistent EV in adults. In some cases, it may completely disappear, or it can show up as a mobile, elongated structure in the RA [1,8]. Furthermore, almost all patients with the persistence of sinus venosus valves have an associated atrial septal defect (ASD) or a patent foramen ovale (PFO) [6]. Thus, symptoms of persistent EV depend on the degree of right-to-left shunting via ASD or PFO.

On an echocardiogram, the EV most commonly appears as a crescentic fold of the endocardium extending from the rim of the IVC. Persistent EV has been identified as an isolated finding in 82% of children [3]. In a study of 306 patients, Schuchlenz et al. [9] divided the sample into two groups: patients with cryptogenic stroke (N = 211) and patients without cerebrovascular events (N = 95). All the patients were investigated by transesophageal echocardiography, color Doppler, and contrast echocardiography. They reported that EVs could be visualized in 57% of the cases. Additionally, 70% of patients with EV had an associated PFO (p < 0.001). The prevalence of PFO in groups 1 and 2 was 61% and 30%, respectively (p < 0.001). It was concluded that EV is a common finding in patients with PFO. This may be due to the redirection of the flow from the RA to the left atrium (LA), which would prevent the spontaneous closure of the foramen ovale after birth. Therefore, it may predispose these patients to an increased risk of paradoxical embolism [9]. Occasionally, a prominent EV may be confused with other pathologies such as cardiac tumors, thrombi, or vegetation [10]. In the majority of cases, the term ‘giant EV’ describes the persistence valve of the systemic venous sinus [4]. Sometimes, persistent EV may be confused with cor triatriatum, cardiac tumors, or thrombus in an echocardiographic examination [10]. Asymptomatic patients with an isolated, persistent EV typically require no further intervention. Meanwhile, very few cases of obstruction in right ventricular (RV) inflow caused by persistent EV have been reported in the literature [4]. Rarely, a prominent EV may cause a significant obstruction of RV inflow, which would require surgery [4,8,11]. Management plans should be individualized on a case-by-case basis, depending on the severity of cyanosis and the degree of RV inflow obstruction [4].

One of the first cases of persistent EV was reported in the literature by Rossall et al. [12]. The patient was a 19-year-old man with a history of varicose veins in the lower limbs over two years. He was found to have hepatomegaly and gynecomastia. After exploratory laparotomy, the final diagnosis was IVC obstruction at the level of hepatic veins. However, about four years later, he was re-admitted with a high fever, remarkable ascites, and bilateral pleural effusion. Despite treatment, he expired shortly after. During the autopsy, it was found that the IVC was almost blocked by an abnormally large EV, which had a cord-like shape. Its size was 4.4 cm in length and 2.6 cm in depth [12]. This case underscored that complicated persistent EV is an under-recognized clinical entity that may be easily missed.

Our patient was a previous marathon runner without any significant personal medical history (PMH). His chief complaint was left flank pain, and his recent history included a passage of renal stones two weeks earlier. A CT scan was indicated due to suspected nephrolithiasis. A calculus (4.8 mm) was detected in the left distal ureterovesical junction, together with mild-moderate hydronephrosis. Another remarkable incidental finding included dilated IVC, renal veins, and iliac veins, extending distally to femoral veins (Figure 3, Figure 4). Related findings in a normal person have been included for comparison [5]. TTE reported a mild left atrial enlargement, a normal ejection fraction (EF), and an estimated pulmonary artery systolic pressure of 65% and 26 mmHg, respectively. A prominent EV was visualized in the RA. Notably, PFO was not completely ruled out due to limited views in TTE. Although the IVC is dilated (3.8 cm), no signs or symptoms suggesting a liver disease, such as jaundice, abdominal pain, or abnormal bleeding. Additionally, echocardiography helped rule out structural heart pathologies or systolic heart failure. However, because we did not conduct a contrast echocardiography in this case, we could not rule out an ASD or PFO. Regarding the association between these pathologies and EV, Morishita et al. [6] described two cases presenting with cyanosis with the underlying pathologies, including persistent EV and ASD. Commonly, cyanosis combined with ASD is almost always associated with an increase in right ventricular pressure, such as pulmonary hypertension. However, in these two cases, the right ventricular pressure was normal. Therefore, cyanosis may be explained by a right-to-left shunt facilitated by a prominent EV, which helps divert the flow from the RA to the LA [6,13].

One more possible implication of persistent EV is that it may help with appropriate planning before interventional procedures. Strotmann et al. [14] reported a case of transcatheter closure of a secundum ASD following a brainstem stroke. The first attempt was not successful because the EV was trapped between the guidewire and the inferior rim of the ASD. The occluding device had to be retracted. The second time was successful without any entrapment of the EV. Afterward, the ASD was closed without any complications. During the bolus of contrast medium, the authors demonstrated that a flow from IVC to RA was redirected towards the fossa ovalis by the EV. Therefore, they suggested that, in this case, the persistent EV may have contributed to the paradoxical cerebral embolism.

Our patient was ultimately discharged without any further intervention. However, due to the potential risk of portal hypertension and liver compromise in the future, we suggested that his future follow-ups should be based on clear communication between all the healthcare providers and the patient to keep everyone well informed about his condition. Management as well as screening plans would be individualized based on his preferences and physicians’ recommendations. Imaging modalities may include a CT scan, echocardiogram, and vascular ultrasound, all of which should be indicated based on his clinical condition. Furthermore, because we could not rule out a PFO or ASD completely, it was strongly suggested that he and his healthcare providers know about this to have an appropriate plan based on the discretion of his physicians.

## Conclusions

Although persistent EV is often a benign structural variant, it may lead to clinical symptoms and serious complications. The management approach should be individualized based on clinical symptoms and the degree of right-to-left shunting. We presented a case with an incidental finding of a dilated IVC and abdominal venous system with the presence of a prominent EV. The recognition of persistent EV on imaging modalities is helpful not only in reaching a correct diagnosis but also in planning various interventions that involve RA. This case also highlighted the importance of an interdisciplinary approach, which may require radiology, vascular surgery, internal medicine, gastroenterology, cardiology, and urology. Finally, the patient was recommended to follow up regularly to be able to detect any potential complications and have them promptly addressed.
